# Long non-coding RNA stabilizes the Y-box-binding protein 1 and regulates the epidermal growth factor receptor to promote lung carcinogenesis

**DOI:** 10.18632/oncotarget.10006

**Published:** 2016-06-14

**Authors:** Ming-Ming Wei, Yong-Chun Zhou, Zhe-Sheng Wen, Bo Zhou, Yun-Chao Huang, Gui-Zhen Wang, Xin-Chun Zhao, Hong-Li Pan, Li-Wei Qu, Jian Zhang, Chen Zhang, Xin Cheng, Guang-Biao Zhou

**Affiliations:** ^1^ State Key Laboratory of Membrane Biology, Institute of Zoology, Chinese Academy of Sciences, Beijing 100101, China; ^2^ Department of Thoracic Surgery, The Third Affiliated Hospital of Kunming Medical University (Yunnan Tumor Hospital), Kunming 650106, China; ^3^ Department of Thoracic Surgery, The Cancer Hospital, Sun Yat-Sen University, Guangzhou 510060, China

**Keywords:** air pollution, lung cancer, lncRNA CAR intergenic 10, YB-1, EGFR

## Abstract

Indoor and outdoor air pollution has been classified as group I carcinogen in humans, but the underlying tumorigenesis remains unclear. Here, we screened for abnormal long noncoding RNAs (lncRNAs) in lung cancers from patients living in Xuanwei city which has the highest lung cancer incidence in China due to smoky coal combustion-generated air pollution. We reported that Xuanwei patients had much more dysregulated lncRNAs than patients from control regions where smoky coal was not used. The lncRNA *CAR intergenic 10* (*CAR10*) was up-regulated in 39/62 (62.9%) of the Xuanwei patients, which was much higher than in patients from control regions (32/86, 37.2%; p=0.002). A multivariate regression analysis showed an association between *CAR10* overexpression and air pollution, and a smoky coal combustion-generated carcinogen dibenz[a,h]anthracene up-regulated *CAR10* by increasing transcription factor FoxF2 expression. *CAR10* bound and stabilized transcription factor Y-box-binding protein 1 (YB-1), leading to up-regulation of the epidermal growth factor receptor (EGFR) and proliferation of lung cancer cells. Knockdown of *CAR10* inhibited cell growth in vitro and tumor growth in vivo. These results demonstrate the role of lncRNAs in environmental lung carcinogenesis, and *CAR10*-YB-1 represents a potential therapeutic target.

## INTRODUCTION

Cancer is the consequence of internal factors such as inherited mutations and external factors such as tobacco smoke and environmental pollution [1]. Approximately 75–90% of cancers have been thought to be caused by environmental pollutants and unhealthy life-styles [2]. In particular, 90% of the lung cancer deaths are caused by cigarette smoke [3], and outdoor and indoor air pollution have been classified as lung carcinogens in humans [4; 5]. Moreover, 80% of the global population resides in locations where the ambient pollutant concentrations exceed the World Health Organization (WHO) Air Quality Guideline [6]. The key carcinogens found in both tobacco smoke and ambient particulate matter (PM) pollution are polycyclic aromatic hydrocarbons (PAHs), a ubiquitous group of environmentally persistent organic compounds of various structures and varied toxicities [3; 7]. PAHs react with DNA to form covalently bound DNA adducts and cause mutations in genes [3]. Lung cancers in smokers have much more somatic genomic mutations than in non-smokers [8], and patients from air polluted regions have much more mutations than patients from control regions [9]. These studies demonstrate that environment-gene interactions play a key role in lung carcinogenesis. However, whether the pollutants modify other molecules such as long non-coding RNAs (lncRNAs) to promote lung cancer, remains unclear.

LncRNAs are a large group of non-coding RNAs with a length of more than 200 nucleotides. As compared with the protein-coding RNAs, lncRNAs are generally poorly conserved in species and expressed at a low and tissue-specific manner in multiple human organs [10]. LncRNAs play tremendous roles in diverse biological processes, such as cell proliferation, differentiation, apoptosis, migration and stem cell pluripotency, through distinct mechanisms at the transcriptional, post-transcriptional or epigenetic levels [10; 11]. Dysregulation of lncRNAs has been shown to affect a broad spectrum of genes by creating complex regulatory networks that are composed of DNA, RNA and proteins [11]. Emerging evidence suggests that abnormalities in lncRNAs play a role in tumorigenesis and are associated with cancer diagnosis, staging, treatment response, metastasis and patient survival. Aberrantly expressed lncRNAs may represent novel biomarkers and therapeutic targets for cancers [12]. In lung cancers, significantly dysregulated lncRNAs have been reported [13], and many of them, e.g., *PANDAR* [14], *DLX6-AS1* [15], *BCYRN1* [16], *HNF1A-AS1* [17], *ANRIL* [18], *MEG3* [19], and others, are involved in lung cancer pathogenesis, cell proliferation, invasion and metastasis, and drug resistance. However, the roles that lncRNAs play in environmental lung carcinogenesis remain unclear, with the identification of lung cancers causally associated with environmental pollution as a prerequisite.

The lung cancer incidence in Xuanwei city of the Yunnan Province is among the highest in China for both males and females. This is attributed to the smoky coal combustion-generated high levels of air pollution [7; 20–23], providing a unique opportunity to dissect environmental lung carcinogenesis. In this city, residents used smoky coal in unvented indoor fire pits for domestic cooking and heating until the 1970s, leading to severe indoor pollution by PM smaller than 2.5 μm in diameter (PM_2.5_)/PM smaller than 10 μm in diameter (PM_10_). Both PM_2.5_ and PM_10_ in Xuanwei contain high concentrations of PAHs [7], similar to PM_2.5_ pollution recently occurred in Beijing and other cities [24]. Coal-burning is also the main source of PM_2.5_ pollution in Beijing [24]. In Xuanwei, near all women cook food on the household stove and are non-smokers, and the female-to-male ratio of lung cancer incidences is 1: 1.09, which is much higher than that of China's national average of 1:2.08 [20]. In this region, smoky coal combustion emission is a significant, while tobacco smoke is a weak and not significant, lung cancer risk [25]. Stove improvements were made in late 1970s, and a reduction in lung cancer incidence was noted in the 1990s, supporting the association between indoor air pollution and lung cancer [22]. However, lung cancer incidence in this city increases in the 2000s, possibly due to outdoor air pollution caused by the coal-burning industrial plants that moved into the area and the improvement in diagnostic approaches [23; 26]. Thus, lung cancer in this highly polluted region (HPR) provides a model to study the interactions between the environment and genetic/epigenetic factors including lncRNAs.

To systematically investigate the environmental lung carcinogenesis, we used Xuanwei lung cancer as a model and analyzed the abnormalities in cancer genomes [9], genome-wide DNA methylation, miRNAs [27] and lncRNAs, and inflammation factors [28]. The abnormalities found in the HPR lung cancers were tested in patients from control regions (CR) where smoky coal was not used, to compare the difference between HPR and CR lung cancer. In this study, we explored the abnormal lncRNAs in non-small cell lung cancers (NSCLCs) from HPR and CR.

## RESULTS

### LncRNA and mRNA microarrays

**Figure 1:**
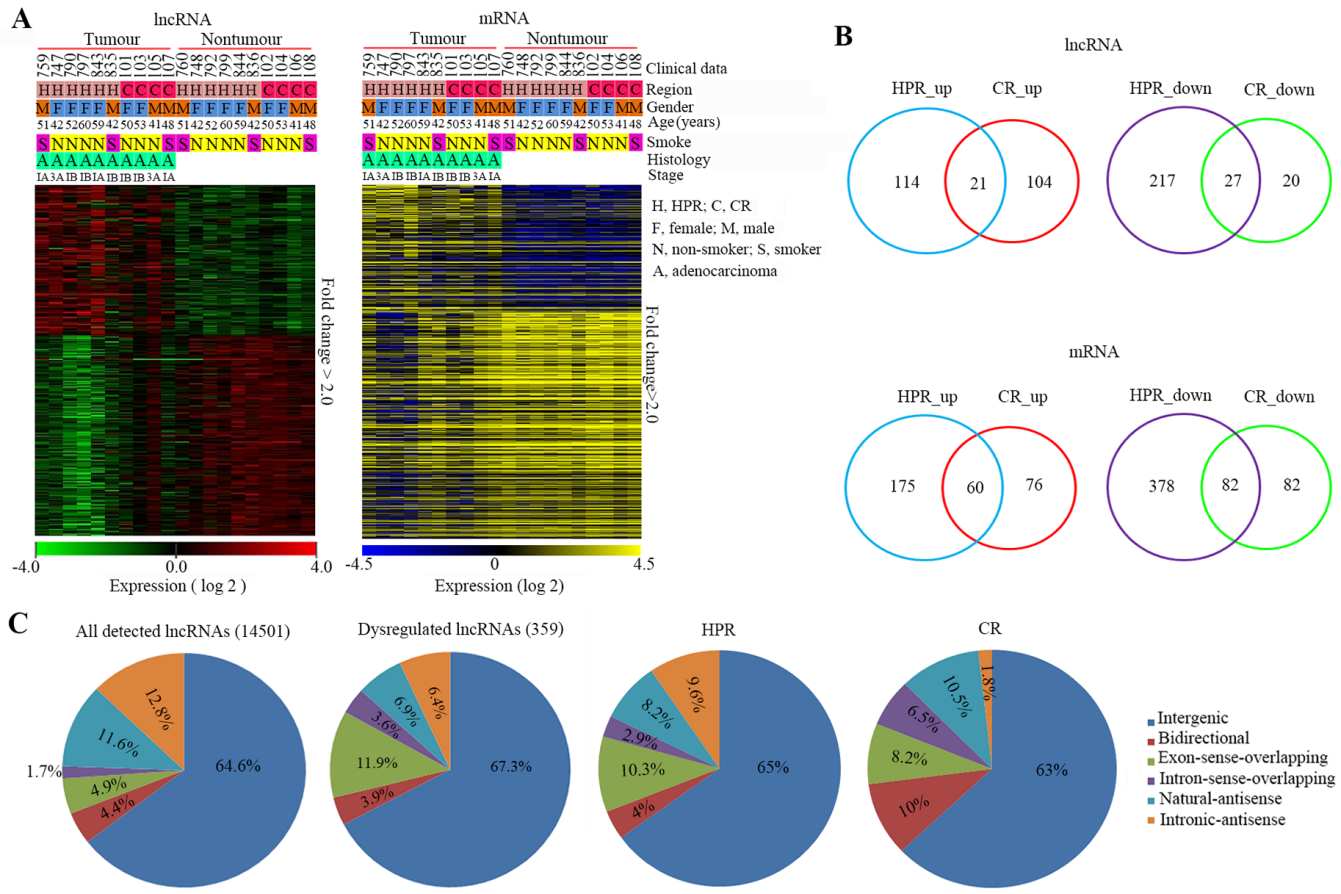
Expression patterns of lncRNAs and mRNAs in HPR and CR NSCLCs **A.** Hierarchical clustering analysis of 359 lncRNAs (154_up and 205_down) and 714 (248_up and 466_down) mRNAs that were dysregulated between 10 NSCLC samples and corresponding normal control samples (Fold-change>2; P < 0.05). **B.** Venn diagrams illustrating dysregulated mRNAs and lncRNAs in HPR and CR NSCLCs, respectively (fold-change>2.0; p<0.05). **C.** Pie charts summarizing the classification of all detected and dysregulated lncRNAs in the 10 NSCLC tissues, and differentially expressed lncRNAs in patients from HRP and CR. LncRNAs were classified as six subgroups according to their relationships with their associated protein-coding genes in the genome.

An Arraystar Human lncRNA Microarray v2.0 (containing 33045 lncRNAs and 30215 mRNAs; Arraystar, Rockville, MD, USA) was used to perform the lncRNA microarray analysis of the tumor tissues and the counterpart adjacent normal lung tissues from ten patients with NSCLCs (6 from HPR and 4 from CR; Figure 1A), and the statistically significant (p<0.05) lncRNAs and mRNAs that showed 2-fold differences between the tumor tissues and paired control tissues were selected for further study. A hierarchical cluster analysis of the differentially expressed lncRNAs and mRNAs revealed distinct expression profiles in the tumor tissues compared to their non-tumor normal controls (Figure 1A and Tables S1 and S2).

### Differential expression patterns of the lncRNAs and mRNAs between the HPR and CR NSCLCs

We compared the lncRNA and mRNA expression profiles of patients from both regions, and found that in the HPR NSCLCs there were 379 lncRNAs and 695 mRNAs, the expression levels of which were significantly altered (Table S3 and S4), while in the CR NSCLCs, only 172 lncRNAs and 300 mRNAs were significantly dysregulated (Table S3 and S4), demonstrating that the HPR patients had more abnormal noncoding and coding RNAs than the CR cases. Compared to their counterpart normal lung tissues, the tumor samples from HPR NSCLCs harbored more down-regulated lncRNAs and mRNAs [244/379 (64.4%) and 460/695 (66.2%), respectively] (Figure 1B). However, the lncRNAs in the CR NSCLCs were mainly up-regulated in tumor samples compared to their counterpart normal lung tissues (125/172, 72.7%; Figure 1B).

Among the lncRNAs detected in this study, the intergenic lncRNAs were the most altered ones (67.3%) in both the HPR and CR NSCLCs (Figure 1C). LncRNAs of exon-sense-overlapping and natural-antisense were also two categories that were altered in both regions (Figure 1C). The intronic-antisense lncRNAs were more frequently altered in the HPR patients, while bidirectional and intron-sense-overlapping lncRNAs were more frequently perturbed in the CR NSCLCs (Figure 1C).

A GO analysis [29] of the dysregulated mRNAs was performed. In all the patients, the up-regulated genes were mainly enriched in vesicle-mediated transport, centrosome separation, and protein modification process, while the down-regulated genes were enriched in vasculature development, response to stimulus, and blood vessel development in all of the patients (Figure S1A, Table S5). In the HPR patients, the up-regulated genes were mainly involved in chromosome organization, chromatin assembly or disassembly, and nucleosome assembly, while the down-regulated genes were involved in vasculature development, angiogenesis, and blood vessel development (Figure S1B). In the CR NSCLCs, the up-regulated genes were mainly involved in vesicle-mediated transport, centrosome separation, and protein modification process, while the down-regulated genes were mainly involved in vasculature development, blood vessel development, and response to steroid hormone stimulus (Figure S1C, Table S5).

### Overexpression of lncRNA *CAR intergenic 10* in lung cancer is associated with air pollution

**Figure 2:**
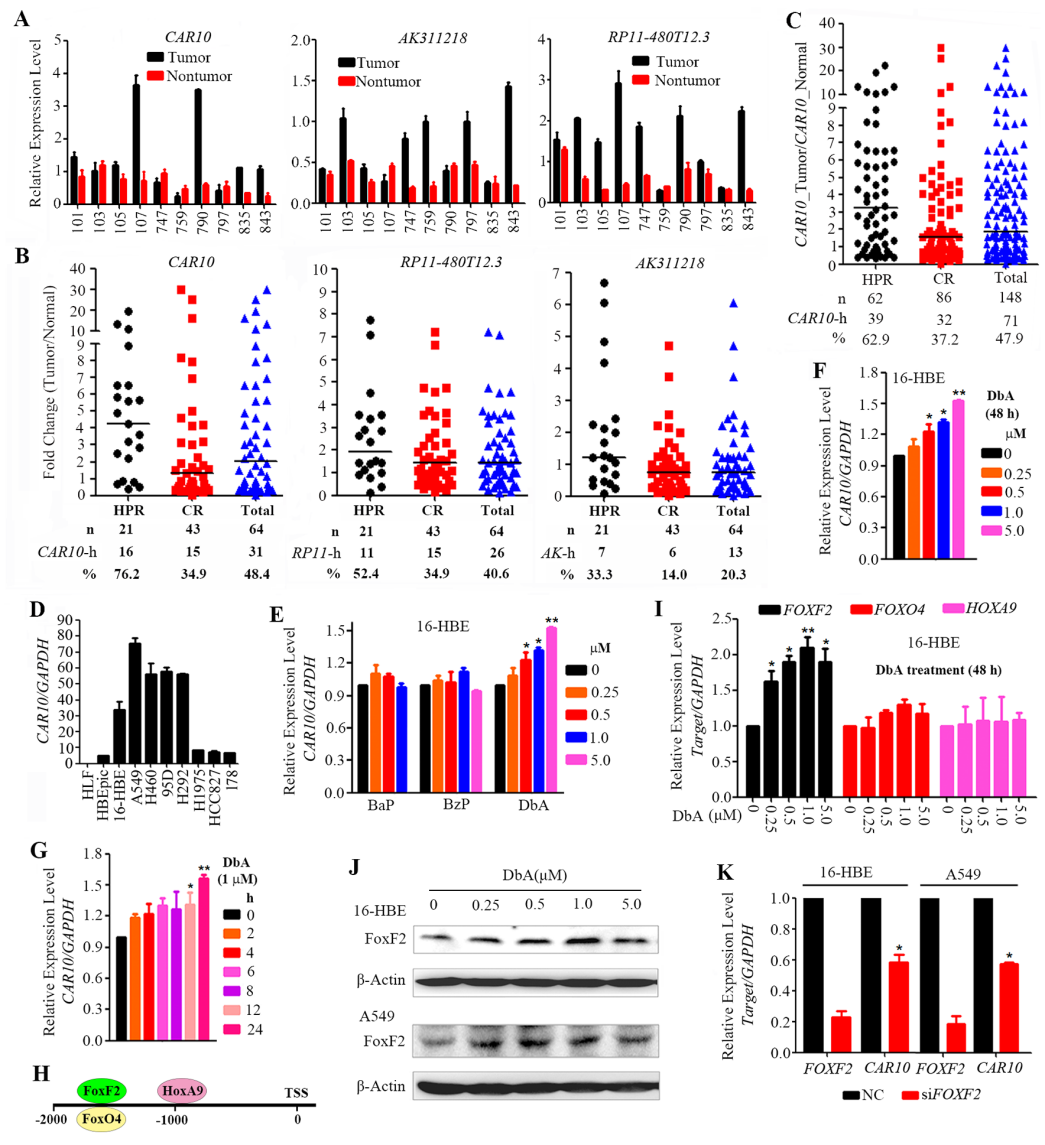
Overexpression of *CAR10* in NSCLCs **A.** The expression of the lncRNAs *CAR10*, *AK311218*, and *RP11-480I12.3* was tested by qRT-PCR in 10 patients whose tissues were analyzed by a lncRNA microarray. **B.** The expression of the 3 selected lncRNAs was tested in 64 NSCLCs. **C.** The expression of *CAR10* was tested in 148 patients by qRT-PCR. **D.** The expression of *CAR10* in several normal or lung cancer cell lines detected by qRT-PCR. **E.** The expression of *CAR10* in 16HBE cells treated with bezo[a]pyrene (BaP), benzo[g,h,i]perylene (BzP) or dibenz[a,h]anthracene (DbA) for 48 hours. Detected by qRT-PCR. **F, G.** The expression of *CAR10* in 16HBE cells treated with DbA at indicated concentrations for indicated time points, detected by qRT-PCR and the relative expression level of *CAR10/GAPDH* was calculated. The error bars indicate the SD of three independent experiments; *P < 0.05, **P < 0.01. **H.** Potential transcription factor binding sites in the *CAR10* promoter. **I.** The relative expression of *FOXF2, FOXO4, and HOXA9* in 16HBE cells treated with DbA at indicated concentrations for 48 hours. The expression of the genes was detected by qRT-PCR. **J.** The expression of FOXF2 in 16HBE and A549 cells treated with DbA at indicated concentrations for 48 hours. The expression of FOXF2 was detected by Western blot using indicated antibodies. **K.** The relative expression of *FOXF2* and *CAR10* in cells transfected with siNC or siFOXF2. The error bars indicate the SD of three independent experiments; *P < 0.05, **P < 0.01.

To identify lncRNAs that are critical to lung carcinogenesis, we selected three lncRNAs, *CAR intergenic 10* (hereafter, *CAR10*), *AK311218*, and *RP11-480I12.3*, from the most up-regulated lncRNAs in the HPR NSCLCs (Table S3) and tested their expression by quantitative reverse transcription polymerase chain reaction (qRT-PCR). The expression of these lncRNAs was consistent with the microarray analysis (Figure 2A). We expanded these observations in additional 64 pairs of tissues (including 21 HPR patients and 43 CR NSCLCs) by qRT-PCR and found that *CAR10* was overexpressed in 16/21 (76.2%) of the HPR and 15/43 (34.9%) of CR NSCLCs (Figure 2B). *RP11-480I12.3* overexpression was seen in 11/21 (52.4%) of the HPR NSCLCs and 15/43 (34.9%) of the CR patients, and overexpressed *AK311218* was seen in 7/21 (33.3%) of the HPR patients and 6/43 (14%) of the CR NSCLCs (Figure 2B).

We therefore selected *CAR10* for further study in a total of 148 pairs of patient samples. *CAR10* was overexpressed in 39/62 (62.9%) of the HPR patients and 32/86 (37.2%) of the CR cases (p=0.002; Figure 2C, Table 1). *CAR10* was also overexpressed in lung cancer cell lines compared to normal human bronchial epithelial cells (HBEpiC and 16HBE) and normal human lung fibroblasts (HLF) (Figure 2D). The multivariate logistic analyses showed that *CAR10*-high was statistically significantly associated with the Xuanwei region (p=0.004), while the association between *CAR10*-high and tobacco smoke or the TNM stages was not significant (Table 2).

**Table 1:** Baseline demographic characteristics of the 148 patients underwent *CAR10* analyses

Characteristics	Cases, n	*CAR 10*-high, n (%)	P values
**Total**	**148**	**71 (47.97)**	
**Area**			
HPR	62	39 (62.90)	0.002
CR	86	32 (37.20)	
**Gender**			
male	89	41 (46.06)	0.568
female	59	30 (50.84)	
**Smoking**			
***Total***			
smoker	66	31 (46.96)	0.830
Non-smoker	80	39 (48.75)	
unknown	2	1 (50.00)	
**CR**			
smoker	40	15 (37.50)	0.958
non-smoker	46	17 (36.96)	
**HPR**			
smoker	26	16 (61.53)	0.801
non-smoker	34	22 (64.70)	
unknown	2	1 (50.00)	
**Age**			
<65	109	54 (49.54)	0.393
>=65	34	14 (41.17)	
unknown	5	2 (40.00)	
**Histology**			
AD	103	53 (51.45)	0.178
SCC	41	16 (39.02)	
LCLC	2	1 (50.00)	
SCLC	1	0	
Carcinoid	1	1	
**Stage**			
IA-IIB	92	49 (53.26)	0.145
IIIA-IV	45	18 (40.00)	
unknown	11	4 (36.36)	

AD: adenocarcinoma; SCC: squamous carcinoma; LCLC: large cell lung cancer; SCLC: small cell lung cancer.

**Table 2:** Multivariate logistic analyses of the association between lncRNA *CAR10* high expression and the clinical characteristics of the patients

Variable	Odds ratio	95.0% Confidence Interval	P values
Total (HPR&CR) patients, n=148			
Region (HPR vs CR)	2.861	1.456-5.624	0.004
Age	1.165	0.568-2.390	0.676
Gender	1.093	0.516-2.316	0.816
Smoking	0.912	0.386-2.150	0.833
Histology	1.255	0.627-2.512	0.521
TNM stage	1.374	0.762-2.478	0.291
HPR patients, n=62			
Age	1.616	0.291-8.975	0.583
Gender	1.220	0.418-3.558	0.716
Smoking	0.478	0.107-2.143	0.335
Histology	0.924	0.193-4.409	0.921
TNM stage	1.864	0.618-5.621	0.269
CR patients, n=86			
Age	1.195	0.534-2.672	0.664
Gender	0.856	0.261-2.813	0.798
Smoking	1.162	0.453-2.980	0.754
Histology	1.278	0.564-2.894	0.557
TNM stage	1.225	0.613-2.448	0.566

To verify the association between environmental pollution and *CAR10* overexpression, we tested the effects of PAH compounds on *CAR10* expression in the normal human lung epithelial cell line 16HBE [30]. To do this, 16HBE cells were treated for 48 hours with bezo[a]pyrene (BaP), dibenz[a,h]anthracene (DbA) and benzo[g,h,i]perylene (BzP) which are found in smoky coal emission [31]. We found that BaP and BzP did not interfere with *CAR10* expression (Figure 2E), whereas DbA significantly up-regulated *CAR10* expression in a dose- (Figure 2F) and time- (Figure 2G) dependent manner.

Next, we investigated how DbA induced the up-regulation of *CAR10*. An analysis of the promoter sequence led to the identification of three potential binding sites for the transcription factors FoxF2 (-1702 to -1689), FoxO4 (-1702 to -1687), and HoxA9 (-994 to -981) upstream of the transcription start site (TSS) of *CAR10* (Figure 2H). By using qRT-PCR, we found that in the 16HBE cells, treatment with DbA induced up-regulation of *FOXF2* in a dose-dependent manner (Figure 2I), whereas the expression of *FOXO4* and *HOXA9* was not altered (Figure 2I). DbA also up-regulated FOXF2 at protein level in 16HBE and A549 cells (Figure 2J). Moreover, silencing of *FOXF2* by specific siRNA resulted in down-regulation of *CAR10* (Figure 2K). These results suggest that *CAR10* is a downstream target of FOXF2 and air pollution may cause up-regulation of *CAR10* at least partially by increasing FOXF2 expression.

### The coding potential of *CAR10* is limited

Using the Open Reading Frame (ORF) Finder (http://www.ncbi.nlm.nih.gov/gorf/gorf.html) and ATGpr [32], we analyzed the sequence of *CAR10* and identified 11 potential ORFs that might code peptides of 35 to 100 amino acids (Figure S2A, S2B). By using the Coding Potential Calculator (CPC) [33], the coding potential of *CAR10* was scored as -0.91, which was much less than that of the protein-coding genes *GAPDH* (14.37) and *ACTA1* (11.48) but was approximately equal to that of the lncRNAs *HOTAIR* (-1.05) and *MALAT1* (0.34) (Figure S2C). Furthermore, the largest potential ORF of *CAR10* was used to test its coding activity. To do so, the CDS sequence of *EGFP* was inserted in-frame into the potential *CAR10* ORF (Figure S2D) and the plasmids were transfected into HEK293T cells. However, the potential EGFP-CAR10 fusion protein was not detected by fluorescence microscopy (Figure S2E) or Western blot (Figure S2F). These data confirmed that *CAR10* has limited protein-coding potential.

### *CAR10* is required for lung cancer cell proliferation

**Figure 3:**
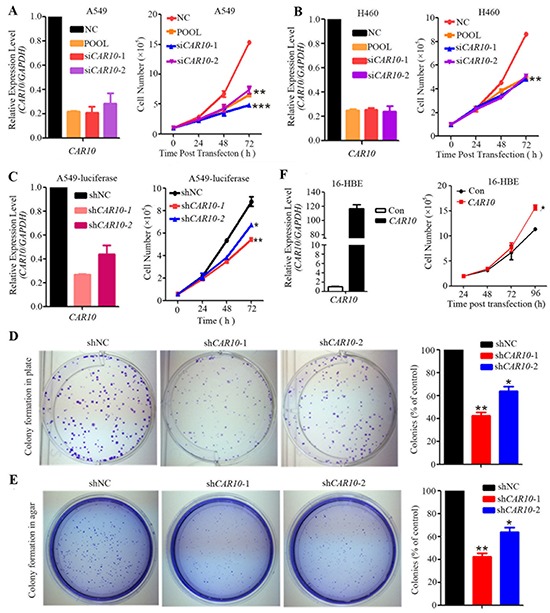
*CAR10* is required for lung cancer cell proliferation **A, B.** A549 (A) and H460 (B) cells were transfected with si*CAR10* and cell proliferation was assessed by trypan blue exclusion analyses. **C.** A549-luciferase cells were transfected with sh*CAR10* and cell proliferation was monitored by the trypan blue exclusion analyses. The relative expression of *CAR10* was detected by qRT-PCR 48 hours after si*CAR10* or sh*CAR10* transfection. **D.** Colony formation assays of plates of A549-luciferase cells stably expressing negative control and *CAR10* shRNAs. **E.** The representative results of the soft-agar assay of A549-luciferase cells stably expressing negative control and *CAR10* shRNAs. **F.** 16HBE cells were transfected with *CAR10*, the cells were harvested and the expression of *CAR10* was tested by qRT-PCR, and the cell number was detected by a trypan blue exclusion analysis. Con, control. The error bars indicate the SD of three independent experiments; *P < 0.05, **P < 0.01.

We investigated the role of *CAR10* in lung cancer pathogenesis by altering its expression in lung cancer lines and 16HBE cells. Two small interference RNAs (siRNAs; Table S6) were used to deplete *CAR10* in NSCLC cell lines. The cell growth curves indicated that cell proliferation was suppressed in A549 (Figure 3A) and H460 cells (Figure 3B) when *CAR10* was knocked down by specific siRNAs (si*CAR10*-1 and -2). A colony forming assay was performed to further assess the effect of *CAR10* on clonogenic activity of lung cancer cells. To do this, A549 cells with a luciferase reporter system (named A549-luciferase) were engineered to stably express control or *CAR10* shRNAs, and the persistent inhibition of *CAR10* resulted in decreased cell growth (Figure 3C). Knockdown of *CAR10* by shRNAs inhibited the clonogenic activity of A549-luciferase cells detected by plate foci formation (Figure 3D) and soft agar (Figure 3E) assays. In contrast, the exogenous expression of *CAR10* accelerated the growth of 16HBE cells compared to cells transfected with the vector control (Figure 3F). These results indicate that *CAR10* plays an important role in lung cancer cell proliferation.

### Identification of *CAR10* binding proteins

**Figure 4:**
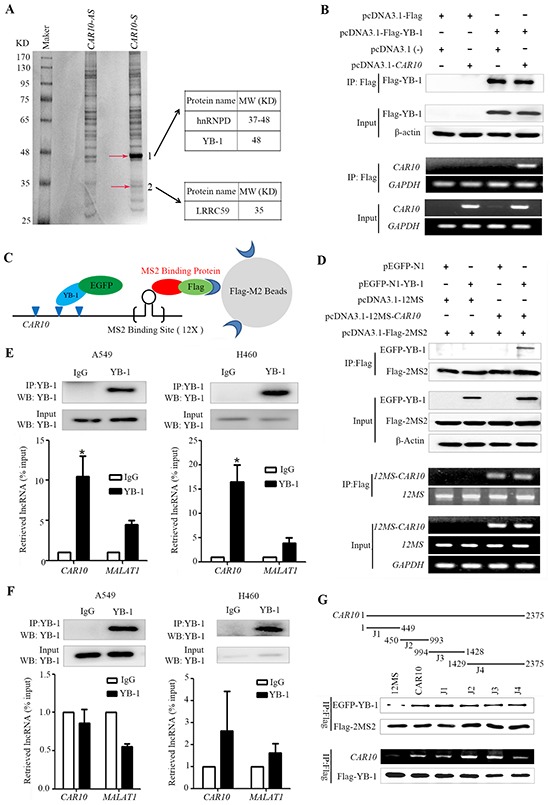
*CAR10* interacts with the transcription factor YB-1 **A.** SDS-PAGE gel of proteins pulled down by biotinylated *CAR10* and an antisense transcript. The bands indicated by arrows were submitted for mass spectrometry analysis. **B.** An RNA immunoprecipitation (RIP) of Flag-YB-1 in HEK293T cells. Flag-YB-1 in the input and IP samples was detected by Western blot (top); CAR10 in the input and IP samples was detected by RT-PCR (bottom). **C.** A schematic representation of the 2MS2-12MS-*CAR10* RNA pull-down system. **D.** The 2MS2-12MS-CAR10 pull-down assay in HEK293T cells transfected with indicated plasmids. Top, YB-1 in input and pull-down samples was detected by Western blot. bottom, the expression of CAR10 in input and pull-down samples was detected by RT-PCR. **E.** RIP of endogenous YB-1 in A549 (left) and H460 cells (right) treated with paraformaldehyde. Top, YB-1 in nuclear extract input or IP samples was detected by Western blot using and anti-YB-1 antibody; bottom, the expression of CAR10 and MALAT1 in the RNA-protein complexes was assessed by qRT-PCR. *P < 0.05. **F.** RIP of endogenous YB-1 in A549 (left) and H460 (right) cells without paraformaldehyde treatment. Analyses of RNA or protein samples was performed as described in (E). **G.** Deletion mapping of the YB-1 binding domain in *CAR10* using the 2MS2-12MS pull-down system. Top, a schematic diagram of the full-length and deleted fragments of *CAR10*; middle, YB-1 in the protein complexes pulled down by different *CAR10* fragments was assayed by Western blot; bottom, the expression of *CAR10* and YB-1 in the RIP complexes were analyzed by RT-PCR and Western blot, respectively. 12MS was as a negative control and *CAR10* was as a positive control. All of the experiments were performed in three independent replicates. The bars indicate the SD. *P < 0.05.

LncRNAs can bind proteins including transcription factors to exert their biological functions [11]. To identify its binding proteins, a biotinylated *CAR10* was synthesized and used for RNA pull-down assays, and the binding proteins were analyzed by mass spectrometry. Interestingly, we identified three proteins, heterogeneous nuclear ribonucleoprotein D (hnRNPD, or AUF1), Y-box-binding protein 1 (YB-1), and leucine-rich repeat containing 59 (LRRC59), were able to bind to *CAR10* (Figure 4A). The YB-1/hnRNPD and YB-1/LRRC59 interactions were shown previously [34; 35], suggesting that *CAR10* may directly or indirectly interact with YB-1 to regulate its biological function.

To validate the direct binding of *CAR10* with YB-1, an RNA immunoprecipitation (RIP) with Flag-M2 beads was performed in HEK293T cells transfected with Flag-YB1 and/or *CAR10*, and the abundance of *CAR10* was measured by RT-PCR. *CAR10* was precipitated and detected in cells co-transfected with *CAR10* and *YB-1* (Figure 4B). An RNA pull down assay was performed using the 2MS2-12MS pull-down system [36] and lysates of 293T cells transfected with EGFP-N1-YB-1 and/or 12-MS-*CAR10*, and the results showed that YB-1 was precipitated by *CAR10*-12MS (Figure 4C, 4D), indicating the interaction between YB-1 and *CAR10*.

To further validate the direct binding between YB-1 and *CAR10*, a RIP assay was conducted using the lysates of NSCLC cells treated with paraformaldehyde [37] and an YB-1 antibody to precipitate *CAR10*, which was then detected by qRT-PCR using specific primers (Table S6). We showed that while *CAR10* was not detected in the immunoglobulin G (IgG)-precipitated samples, it was significantly up-regulated in the YB-1-precipitated samples (Figure 4E). However, the negative control lncRNA *MALAT1* was not significantly increased in the YB-1-precipitated samples (Figure 4E). We performed a RIP using the lysates of NSCLC cells that did not receive paraformaldehyde treatment, and the results showed that neither *CAR10* nor *MALAT1* was enriched in the YB-1-antibody-bound complex (Figure 4F), suggesting that YB-1 does not bind to *CAR10* after the cells were lysed. Deletion-mapping of the YB-1 binding site was performed in *CAR10* using the 2MS2-12MS pull-down system and RIP, and the results showed that both the N- and C-terminals of *CAR10* could bind to YB-1, and that the binding affinity of the N-terminal to YB-1 was slightly higher than the C-terminal (Figure 4G).

### *CAR10* protects YB-1 from proteasomal degradation

**Figure 5:**
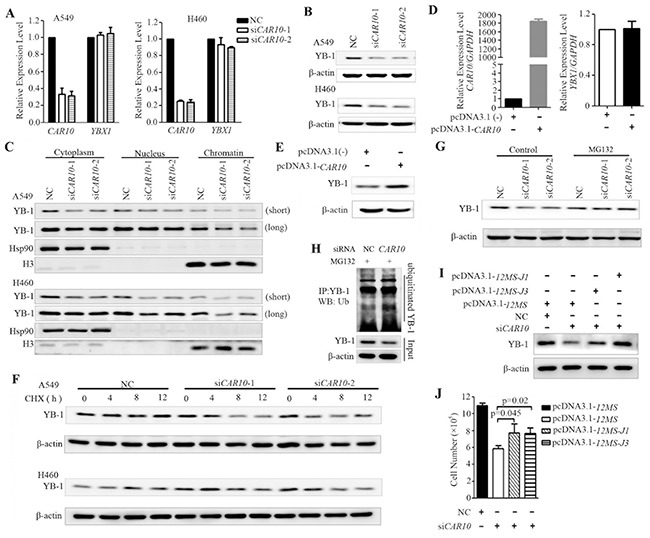
*CAR10* determines the protein stability of YB-1 **A–C.** A549 and H460 cells were transfected with si*CAR10* for 48 hours, the mRNA levels of *CAR10* and *YB-1* were detected by qRT-PCR (A) and YB-1 protein in the whole cell lysate (B) or indicated fractions of the cells (C) was assessed by Western blotting. Hsp90 and H3 were used as the cytoplasmic and chromatin-bound loading controls, respectively. Long, long exposure; short, short exposure. **D, E.** 16-HBE cells were transfected with full-length *CAR10* for 48 hours, and the expression of *CAR10* and YB-1 was evaluated by qRT-PCR (D) or Western blotting (E), respectively. **F.** The protein level of YB-1 in A549 (upper panel) and H460 (lower panel) cells treated with cycloheximide (CHX, 100 μg/ml) in the presence or absence of si*CAR10*. **G.** Western blot analysis of the YB-1 expression in A549 cells transfected with si*CAR10* in the presence or absence of MG132. **H.** A549 cells transfected with si*CAR10* were treated with MG132, lysed, YB-1 protein was immunoprecipitated with an anti-YB-1 antibody, and ubiquitinated YB-1 was detected by using an anti-ubiquitin antibody. **I, J.** A549 cells were transfected with si*CAR10* and then the exogenous 12MS-*CAR10*-J1 or 12MS-*CAR10*-J3 plasmids (see Figure 4G) for 48 hours, and cells were lysed, the proteins were isolated, and Western blot analyses were conducted (I). Cell proliferation was assessed by a trypan blue exclusion analysis (J).

We investigated the effects of *CAR10* on YB-1 function by knockdown or forced expression of *CAR10*. In A549 and H460 cells transfected with *CAR10*-specific siRNA, YB-1 expression was significantly down-regulated at protein but not mRNA level (Figure 5A, B). YB-1 was distributed in the cytoplasm and nucleus of the cells [38], and the nuclear YB-1 expression was a negative prognostic marker of NSCLC [39]. We detected YB-1 expression in the cytoplasm, the soluble nuclear fraction, and the insoluble chromatin-bound fractions of *CAR10*-knockdown cells and found a marked decrease of YB-1 in the insoluble chromatin-bound fraction (Figure 5C). YB-1 expression in the cytoplasm and the soluble nuclear fractions was also reduced (Figure 5C). On the other hand, the exogenous expression of *CAR10* increased YB-1 expression at protein but not mRNA level (Figure 5D, E). These observations demonstrate that *CAR10* up-regulates YB-1 at protein but not mRNA level.

To further explore the mechanism of *CAR10* in up-regulation of YB-1 expression, the A549 and H460 cells were treated with the protein synthesis inhibitor cycloheximide (CHX, 100 μg/ml) in the presence of negative control siRNA (siNC) or si*CAR10*, and the expression of YB-1 was assessed by Western blot. We found that when *CAR10* was not silenced, YB-1 was stable and its expression was not decreased within 12 hours (Figure 5F). However, YB-1 was down-regulated in 4 to 12 hours when the cells were treated with CHX in the presence of si*CAR10* (Figure 5F). YB-1 was degraded by the 20S proteasome in a ubiquitin- and ATP-independent manner and was abolished by the association of YB-1 with messenger RNA [40]. We found that in the presence of the proteasome inhibitor MG132, YB-1 expression in the *CAR10* knockdown cells was markedly increased and reached a level that was comparable to that in the siNC-treated cells (Figure 5G), but the ubiquitinated YB1 was not markedly increased (Figure 5H). In addition, in A549 cells transfected with si*CAR10*, the exogenous expression of the J1 and J3 fragments of *CAR10* (Figure 4G) led to an increase in YB-1 protein expression (Figure 5I). Moreover, while the silencing of *CAR10* by siRNA inhibited A549 cell growth, the transfection of *CAR10* J1 and J3 fragments antagonized this effect (Figure 5J). These observations indicate that *CAR10* is important for maintaining the protein stability of YB-1.

### *CAR10* regulates the expression of epidermal growth factor receptor (EGFR) by stabilizing the nuclear YB-1 protein

**Figure 6:**
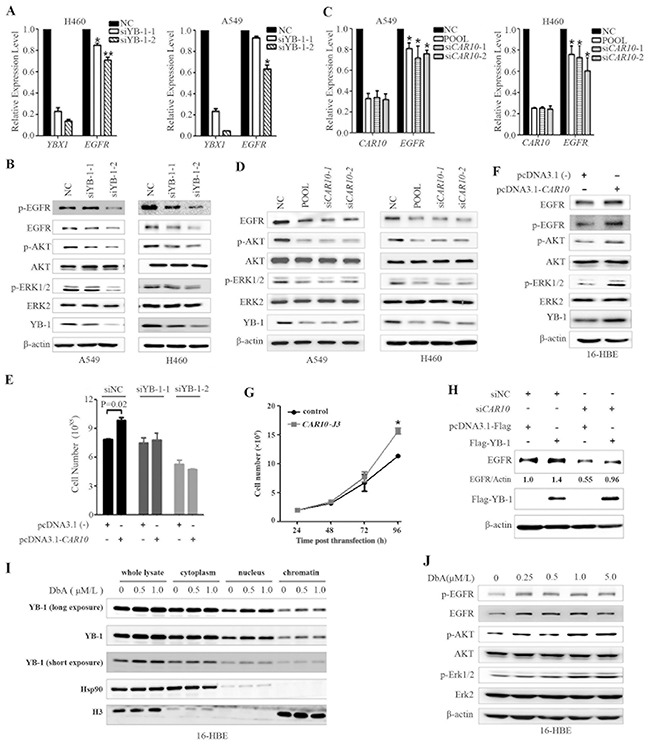
*CAR10* regulates EGFR by stabilizing YB-1 **A, B.** Silencing of YB-1 by specific siRNA resulted in the down-regulation of EGFR at both mRNA (A) and protein (B) levels in H460 and A549 cells as detected by qRT-PCR and Western blotting, respectively. **C, D.** *CAR10* inhibition by specific siRNA also resulted in the down-regulation of EGFR at both mRNA **(C)** and protein **(D)** levels in the cells as detected by qRT-PCR and Western blotting, respectively. **E.** 16HBE cells were transfected with si*YB-1* and/or *CAR10* plasmid, and 48 hours later the cells were analyzed by a trypan blue exclusion analysis. **F.** 16-HBE cells were transfected with plasmid carrying *CAR10* for 48h, lysed, and subjected to Western blot using indicated antibodies. **G.** The growth curve of 16HBE cells transfected with CAR10 J3. *P*=0.02. **H.** A549 cells were transfected with si*CAR10* and/or Flag-*YB-1*, incubated for 48 hours, and lysed for Western blot assay using indicated antibodies. The numbers under the EGFR bands are the expression values relative to Actin as determined by a densitometry analysis. **I.** 16HBE cells were treated with DbA for 48 hours, lysed, proteins of indicated fractions were harvested, and subjected to Western blot using indicated antibodies. **J.** 16HBE cells were treated with DbA for 48 hours, lysed, and subjected to Western blot using indicated antibodies.

YB-1 regulates many genes that are involved in the cell cycle, apoptosis, or drug resistance [41]. Nuclear YB-1 localization is associated with the expression of EGFR, and YB-1 can bind to the promoter of EGFR and regulate its transcription [42]. The specific siRNA-mediated silencing of YB-1 resulted in the down-regulation of EGFR at mRNA (Figure 6A) and protein (Figure 6B) levels. Interestingly, si*CAR10* treatment also induced down-regulation of EGFR at mRNA (Figure 6C) and protein (Figure 6D) levels. In A549 and H460 cells, knockdown of *YB-1* (Figure 6B) or *CAR10* (Figure 6D) led to a decrease in phosphorylated AKT (pAKT) and ERK (pERK). On the contrary, the exogenous expression of *CAR10* promoted cell proliferation (Figure 6E) and up-regulated EGFR at protein level (Figure 6F), and increased pEGFR, pAKT, and pERK (Figure 6F). Transfection of *CAR10-J3* also promoted 16HBE cell growth (Figure 6G). However, when YB-1 was silenced by specific siRNAs, the forced expression of *CAR10* failed to promote cell growth (Figure 6E). In A549 cells, si*CAR10* induced a down-regulation of EGFR while the ectopic expression of YB-1 antagonized this effect (Figure 6H). These results suggest that YB-1 mediates the effect of *CAR10* on EGFR.

We further showed that DbA treatment in 16HBE cells up-regulated the expression YB-1 in total cell lysate and in nucleus and chromatin fractions (Figure 6I). DbA also increased the expression EGFR/pEGFR as well as pAKT and pERK in 16HBE cells (Figure 6J). These results suggest the role of YB-1/EGFR in air pollution-related lung carcinogenesis.

### Down-regulation of *CAR10* inhibits tumor growth *in vivo*

**Figure 7:**
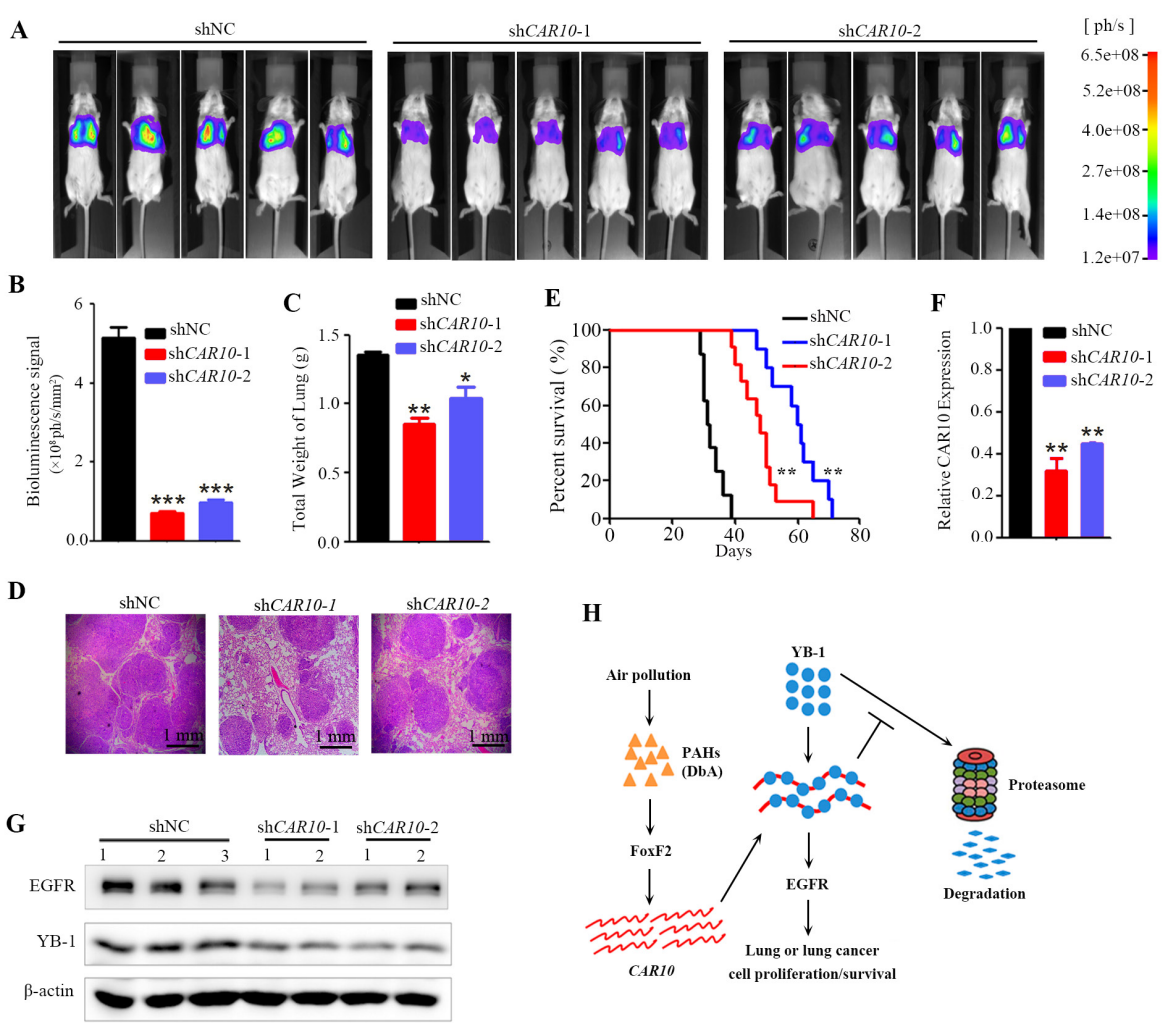
Inhibiting *CAR10* suppresses tumor growth *in vivo* **A.** SCID mice were injected with 1×10^6^ A549-Luciferase cells (transfected with shNC or shCAR10) via tail vein, and 25 days later the mice were detected by the IVIS Spectrum system. **B.** The relative luciferase intensity in the mice. n=15 for each group. **C.** Weight of the lungs from each group of the mice. The error bars indicate the SD; *P < 0.05, **P < 0.01. **D.** Hematoxylin-eosin staining of the lung sections from each group of mice. **E.** Kaplan–Meier survival curve of the mice. n=12 for each group. **F.** The expression of *CAR10* in the tumor samples from the mice of each group. **G.** Western blot analyses of EGFR and YB-1 in tumors from mice of each group. **H.** Schematic representation of *CAR10* in environmental lung carcinogenesis.

To explore the role of *CAR10* in promoting lung cancer growth *in vivo*, A549-luciferase cells stably expressing sh*CAR10* were inoculated into SCID-beige mice via tail vein, and twenty five days later the bioluminescence signal was detected. Interestingly, we found that the luciferase signal in *CAR10* knockdown groups was significantly lower than in the control group (Figure 7A, B), and the weights of the lungs from the control group were substantially heavier than those of the *CAR10* knockdown groups (Figure 7C). These results indicated that the tumorigenesis of the A549 cells was inhibited by *CAR10* silencing. The lung tissue sections from each group were stained with hematoxylin-eosin (H&E) to assess tumor content, and the results showed that the lungs from the control group were almost full of tumor cells, but lungs from the *CAR10* knockdown mice had markedly less tumor cells (Figure 7D). Moreover, the overall survival of the mice harboring the *CAR10* knockdown cells was significantly longer than the control group (Figure 7E). The expression level of *CAR10*, EGFR, and YB-1 was tested in the tumor tissues by qRT-PCR or Western blot, and the results showed that in the lungs of the mice inoculated with sh*CAR10*-A549-luciferase cells, the expression of *CAR10* (Figure 7F), EGFR and YB-1 (Figure 7G) was markedly lower than the control group. These findings demonstrated that knockdown of *CAR10* suppresses lung cancer cell growth *in vivo* by regulating YB-1-EGFR signaling.

## DISCUSSION

Lung cancer is the leading cause of cancer death worldwide, with an estimated 1.8 million new cases and 1.59 million deaths from lung cancer in 2012 [43]. Cigarette smoke and air pollution represent two major causes of lung cancer [3–5], both of which contain a large amount of chemicals such as PAHs that are harmful to humans [44]. Chemical analyses show that organic matter constitutes a major fraction of the total PM_2.5_, followed by sulfate, nitrate, ammonium, elemental carbon and chloride, and PAHs are the main carcinogens of PM_2.5_/PM_10_ [24]. These carcinogens induce genomic mutations, modify gene expression profiles, modulate miRNAs, and trigger chronic inflammation to promote lung cancer [8; 9; 28; 45; 46]. However, our knowledge about environmental lung carcinogenesis remains limited.

To investigate the roles of lncRNAs in environmental carcinogenesis, we used HPR lung cancer samples and microarrays to screen for abnormal lncRNAs, and found that the HPR and CR NSCLCs exhibited differential lncRNA expression profiles (Figure 1). The HPR lung cancers had more altered lncRNAs than the CR cases; in particular, the HPR patients harbored more down-regulated lncRNAs than the CR lung cancers (Figure 1). In expanded validation experiments, we showed that *CAR10* was overexpressed in 39/62 (62.9%) of the HPR NSCLCs and 32/86 (37.2%) of the CR patients (p=0.002; Table 1, Figure 2), and a multivariate logistic analysis showed an association between *CAR10*-high expression and the air-polluted region Xuanwei (p=0.004; Table 2). *CAR10* overexpression was not associated with cigarette smoke in the CR NSCLCs (Table 2), suggesting that the concentration of DbA in tobacco might be less than that in HPR ambient PM. *CAR10* up-regulated the expression of YB-1 by the direct binding and inhibition of its proteasomal degradation, resulting in an up-regulation of EGFR/pEGFR, pAKT and pERK, and a promotion of cancer cell proliferation *in vitro* and *in vivo* (Figure 3 to 7). These results indicate that lncRNAs play an important role in environmental lung carcinogenesis. Since air pollution in Xuanwei is similar to that in Beijing in the carcinogens contained and the source of particulate matter (coal burning), the results in Xuanwei lung cancer may reflect the lung carcinogenesis of Beijing PM_2.5_ pollution.

PAHs are pervasive environmental pollutants that are found ubiquitously, not only in all forms of different environmental media (such as air, soil, and water), but also in various foods that we encounter in our everyday life [47; 48]. PAHs are released into the environment from both natural and anthropogenic sources, and the anthropogenic sources include the exhaust of motor vehicles, petroleum refineries, heating in power plants, combustion of refuse, deposition from sewage, oil/gasoline spills, tobacco smoke, barbeque smoke, and coke production. DbA, a crystalline aromatic hydrocarbon consisting of five fused benzene rings, is carcinogenic and can induce carcinomas in mice. DbA induces DNA damage and gene mutations in bacteria as well as gene mutations and transformation in several types of mammalian cell cultures [49]. However, the tumorigenic mechanism of DbA remains largely unknown, and its effect on lncRNA expression has not been reported. Here we showed that DbA induced the up-regulation of *CAR10* (Figure 2) and EGFR and its downstream signaling molecules (Figure 6J) through the induction of the FoxF2-YB-1 signal cascade (Figure 7H), contributing to lung cancer cell proliferation *in vitro* and *in vivo* (Figure 4 to 7). These results demonstrated that the ubiquitous carcinogen PAH interferes with the expression of lncRNA, and the carcinogen-lncRNA interactions may play an important role in lung carcinogenesis.

*CAR10* is located on chromosome 10 and is flanked by the *fibronectin type III and ankyrin repeat domains 1* (*FANK1*) and *ADAM metallopeptidase domain 12* (*ADAM12*) genes [50; 51]. We investigated the biological function of *CAR10* by assessing its binding proteins, and found that *CAR10* directly interacted with the transcription factor YB-1 in NSCLC cells (Figure 4). A previous study showed that YB-1 had a high affinity for a variety of DNAs and RNAs and had a tendency to bind to sequences rich in A and C [35]. We found that *CAR10* bound YB-1 mainly through its N-terminal sequences (J1 to J3), and *CAR10* binding improved the stability of YB-1 by preventing its proteasomal degradation, leading to the accumulation of this transcription factor in the chromatin fraction as well as the nuclear and cytoplasmic fractions (Figure 5). YB-1 binds to the enhancers of the *EGFR* and regulates its transcription [42]. We found that the overexpression of *CAR10* resulted in up-regulation of EGFR and lung epithelial cell proliferation, whereas si*CAR10* led to EGFR down-regulation and the inhibition of cell proliferation (Figure 5, 6). Previous study showed that *CAR10* positively regulates the transcription of oncogene *ADAM12* by establishing active chromatin structures [50; 51]. Overexpression of *CAR10* may also lead to up-regulation of *ADAM12* to promote air pollution-induced lung carcinogenesis. Thus, our results demonstrated the critical role of *CAR10* in lung cancer pathogenesis.

YB-1 regulates cellular signaling pathways within each of the hallmarks of cancer proposed by Hanahan and Weinberg [52; 53]. For example, it modulates proliferation pathways, overrides cell-cycle check points, promotes replicative immortality and genomic instability, promotes angiogenesis, facilitates invasion and metastasis, and promotes inflammation. Moreover, YB-1 is an important transcription factor that regulates the expression of EGFR, which represents one of the most important therapeutic target for NSCLCs [54], and nuclear YB-1 expression is a negative prognostic marker and is associated with drug-resistance in NSCLCs [39]. Therefore, YB-1 is an attractive therapeutic target for lung cancer. Several approaches have been developed to target YB-1, including the direct targeting of YB-1 using cell-permeable inhibitory peptides, YB-1 siRNAs or oligonucleotide decoys, and indirect inhibition by blocking molecules that activate it [52]. Because *CAR10* was required for YB-1 stability, targeting *CAR10* would result in the proteolysis of this oncoprotein. Hence, *CAR10* silencing by specific siRNA may represent a novel YB-1 inactivating approach. Moreover, the combinatory effects of *CAR10*-YB-1-targeting and EGFR inhibition warrant further investigation.

The identification of the population with a higher risk of lung cancer from the residents of air polluted regions and the 1.4 billion worldwide smokers is critical for lung cancer prevention and treatment outcome. The metabolites of nicotine, NNK, and PAHs are related to lung cancer [3], and a chemokine CCL20 may also be associated with lung cancer risk in smokers [46]. However, a precise prediction approach remains an urgent need to identify those populations. *CAR10* was induced by the PAH compound in 16HBE cells (Figure 2), suggesting that it may have potentials in prediction of lung cancer risk in air polluted region residents, and this possibility warrants further investigation.

## MATERIALS AND METHODS

### Patients and tissue samples

The use of the samples was approved by the Institutional Review Board of the Institute of Zoology, Chinese Academy of Sciences and the local research ethics committees of all participating hospitals. The methods were performed in accordance with the approved guidelines. The diagnosis of lung cancer was confirmed by at least 2 pathologists, and the tumor tissues and adjacent normal lung tissues were obtained with informed consent from 148 patients at local hospitals. The HPR patients enrolled met the following criteria: (1) the patients were residents of Xuanwei where smoky coal was used; (2) the patients resided in their communities and never stayed in other regions for a long period of time (6 months or more); (3) the patients had previously untreated primary lung cancer; and (4) the patients' tissue samples were taken at the time of surgery and quickly frozen in liquid nitrogen. The tumor samples contained a tumor cellularity greater than 60% and the matched control samples had no tumor content. The clinical and pathological data for these patients are shown in Table 1 and Figure 1A.

### RNAs and assays

RNA was isolated using a RNA/DNA midi Kit (QIAGEN, Valencia, CA, USA) or the Trizol reagent (Invitrogen, Frederick, MD, USA). Real-time PCR was performed using the primers listed in Table S6, and lncRNA microarray analysis was conducted according to manufacturer's instructions (Arraystar, Rockville, MD, USA). The RNA pull-down and RNA-immunoprecipitation (RIP) assays were performed as described in detail in the Supplementary Materials and Methods.

### Statistical analysis

All statistical analyses were conducted using SPSS 17.0 software for Windows (Chicago, IL). Statistically significant differences were determined by Student's *t*-test, Wilcoxon rank sum test, or multivariate logistic analysis. The survival curves of mice were constructed according to the Kaplan-Meier method and compared with the log-rank test. *P* values less than 0.05 were considered statistically significant in all cases.

## SUPPLEMENTARY MATERIALS AND METHODS, TABLES AND FIGURES












