# Supplementary material for: Long non-coding RNA stabilizes the Y-box-binding protein 1 and regulates the epidermal growth factor receptor to promote lung carcinogenesis
Source: Oncotarget. 2016 Jun 14;7(37):59556–71. doi: 10.18632/oncotarget.10006 (PMC5312331; doi:10.18632/oncotarget.10006)
Supplement: Supplementary file 1 [file oncotarget-07-59556-s001.pdf]

Long non-coding RNA stabilizes the Y-box-binding protein 1 and regulates the epidermal growth factor receptor to promote lung carcinogenesis

SUPPLEMENTARY MATERIALS AND METHODS

Cell cultures

A549, NCI-H460, HEK293T, and 16HBE [1] cells were cultured in DMEM or RPMI1640 medium supplemented with 10% fetal bovine serum (Hyclone, Logan, UT, USA) in the absence or presence of DbA.

Cell cultures and cell proliferation assays

The human lung adenocarcinoma cancer cell line A549, large cell lung cancer line NCI-H460, and human embryonic kidney cell line HEK 293T were obtained from the American Tissue Culture Collection (ATCC, Manassas, VA, USA). Human normal bronchial epithelial cell line 16HBE [1] was obtained from Clonetics (Walkersville, MD, USA). A549 and HER293T cells were cultured in DMEM medium (Hyclone, Logan, UT, USA) supplemented with 10% fetal bovine serum (Hyclone). H460 and 16HBE cells were cultured in RPMI1640 medium (Hyclone) supplemented with 10% fetal bovine serum. All cells were cultured at 37 °C in 5% (v/v) CO_2 incubator in the presence or absence of DbA. 1.0×10^5 A549 cells and H460 cells were plated in 6-well plates, and treated with siNC and si*CAR10*. Transient transfection of plasmids or siRNAs was conducted using the Lipofectamine 3000 kit (Invitrogen), and *CAR10* shRNAs was transfected with lentiviruses. The numbers of cells were counted at 24, 48, and 72 hours after transfection. 5.0×10^5 16HBE cells were plated in 6-well plates, and were transfected with pcDNA3.1(-) and pcDNA3.1-*CAR10* plasmids. Cells were renewedly plated in 6-well plates at 24 hours post transfection, and the cell numbers were counted at 72 hours post transfection. For rescue assay, cells were plated in 6-well plates after transfection, and counted at 72 hours post transfection. Cell proliferation/growth was evaluated by trypan blue exclusion assay or colony forming assay, and the cell proliferation curves were plotted using the numbers of cells at each time point.

RNA extraction, RT-PCR and real-time PCR

Cell total RNA was isolated using Trizol reagent (Invitrogen, Frederick, MD, USA). Tissues total RNA was extracted with RNA/DNA midi Kit (QIAGEN, Valencia, CA, USA). First-strand cDNA was generated using the M-MLV Reverse Transcriptase (Promega, Fitchburg, WI, USA) and either Oligo (dT) primers or random primers (Takara Biotechnology, Dalian, China). Real-time PCR was performed in the StepOne™ Real-Time PCR System (CFX96; Bio-Rad, Hercules, CA, USA) using SYBR® Premix Ex Taq™ (Takara Biotechnology). RT-PCR was performed with rTaq DNA polymerase (Takara Biotechnology) according to the manufacturer's instructions. The sequences of primers were listed in Table S6.

Microarray analysis

Tumor and paired normal lung tissues harvested from 10 NSCLCs were used for lncRNA microarray analysis. Briefly, 1 μg total RNA from each sample was amplified and transcribed into fluorescent cRNA along with the entire length of the transcripts without 3' bias utilizing a random priming method. The labeled cRNAs were hybridized onto the Human LncRNA Array v2.0 (8 x 60K, Arraystar, Rockville, MD, USA). After being washed, the arrays were scanned with the Agilent Scanner G2505B (Agilent Technologies, Santa Clara, CA, USA). Agilent Feature Extraction software (version 10.7.3.1) was used to analyze acquired array images. Quantile normalization and subsequent data processing were performed using the GeneSpring GX v11.5.1 software package (Agilent Technologies). The differentially expressed lncRNAs and mRNAs with statistical significance were identified using the volcano plot filtering. The threshold we used to screen dysregulated lncRNAs and mRNAs was fold change ≥ 2 and a p-value ≤ 0.05.

Vectors and transfection

The full-length *CAR10* was amplified by PrimerSTAR Max DNA Polymerase (Takara Biotechnology) and sub-cloned into the NheI and Hind III sites of pcDNA3.1 (-) vector (Invitrogen) and pGEM-T easy vector (Promega). The cDNA sequence of EGFP with KOZARA motif was amplified from pEGFP-N1 vector and sub-cloned into the NheI and Hind III sites of pcDNA3.1 (-) vector. The cDNA sequence of EGFP was inserted into the ApaI site of pcDNA3.1-*CAR10* plasmids without frame shift, and named pcDNA3.1-*CAR10-EGFP*. The full-length of *CAR10* was inserted into the EcoR V and Hind III sites of pcDNA3.1-12MS plasmid to obtain the pcDNA3.1-*12MS-CAR10* plasmid. The *CAR10* deletion fragments were sub-cloned into the EcoR V site of the pCDNA3.1-*12MS* plasmid by One Step Cloning Kit (Vazyme™, Nanjing, Jiangsu, China). The CDS sequence of

YB-1 was cloned into the Hind III and BamH I sites of pcDNA3.1-Flag and pEGFP-N1 vector. Transient transfection of plasmids or siRNAs was conducted using the Lipofectamine 3000 kit (Invitrogen) according to the manufacturer's instructions. The double-stranded siRNAs and their negative control siRNAs (siNCs) (GenePharma, Shanghai, China) were transfected into cells at a final concentration of 50 nM. The cells were harvested at 24, 48 or 72 hour after transfection according to experimental needs.

The design of shRNAs was assisted by the use of a web-based software provided by Invitrogen (http://rnaidesigner.invitrogen.com/rnaiexpress/), and the sequences were listed in Table S6. After annealing, double-strand oligos were inserted to the liner lentiviral vector pLKO.1, which was kindly provided by Prof. Wanzhu Jin at the Institute of Zoology, Chinese Academy of Sciences. pLKO.1 shRNA plasmid, psPAX2 packaging plasmid and pMD2.G envelope plasmid were co-transfected into the HEK293FT cells to produce lentivirus with control and *CAR10* shRNAs. Infectious lentiviruses were harvested at 36 hours post transfection and filtered through 0.45 μm PVDF filters and named shNC, sh*CAR10-1* and sh*CAR10-2*. A549-luciferase cells were infected with lentivirus in DMEM containing 8 μg/ml polybrene (Sigma-Aldrich, St. Louis, MO, USA), and selected with 1 μg/ml puromycin (Amresco, Solon, OH, USA) for four weeks. The expression of *CAR10* in the selected cells was tested by qRT-PCR.

Proteins and western blotting

The cells and tissues were lysed in RIPA buffer supplemented with protease inhibitors cocktail (Sigma). Subcellular fractionation proteins were extracted with nuclear and cytoplasmic Protein Extraction Kit (Beyotime, Haimen, Jiangsu, China) according to the manufacturer's instructions. The proteins were electrophoresed by SDS-PAGE under denaturing conditions and transferred onto the nitrocellulose membranes (Millipore Corporation, Darmstadt, Germany). Membranes were blocked in 5% non-fat milk (Thermo Fisher Scientific, Basingstoke, UK) and then incubated with primary antibodies. After being washed, the membranes were incubated with secondary antibodies, and detected by Luminescent Image Analyzer LSA 4000 (GE, Fairfield, CO, USA). Antibodies used in this study were: anti-YB-1, anti-EGFR, anti-AKT, anti-p-AKT (Santa Cruz Biotechnology, Santa Cruz, CA, USA), anti-Hsp90, anti-ERK, anti-p-ERK (CST, Beverly, MA, USA), anti-EGFP, anti-H3 (Abcam, Cambridge, MA, USA), anti-Flag and anti- β-actin (Sigma).

RNA pull-down assay

The full-length *CAR10* was cloned into the pGEM-T easy Vector (Promega). Biotin-labeled *CAR10*-sense and *CAR10*-antisense were transcribed in vitro using a biotin labeling dNTP mix (Roche, Basel, Switzerland) and T7 polymerase (Promega) from linearized pGEM-T-*CAR10* plasmid following the manufacturer's instructions. A549 cells (1.5×10^7) were collected for each group and washed with DEPC-PBS for three times. The cell pellets were re-suspended in 1 ml ice-cold cytoplasm isolation buffer (10 mM Hepes, 1.5 mM $MgCl_2$, 10 mM KCl, 0.5 mM DTT, 0.05% NP-40) and vortexed at highest speed for 10 sec, followed by incubating on ice for 1 min. Nuclei were harvested at 1000 g for 3 min at 4°C. Pellets were re-suspended in 1 ml freshly prepared lysis buffer (50 mM Tris PH7.4 ; 150 mM NaCl, 1% NP-40, 0.5% Triton x-100, 10% glycerol, 1 mM PMSF, 1 mM cocktail, 200U/ml RNaseOUT) and lysed with sustaining vortex for 30 min at 4°C . The nuclear lysates were obtained at 16,000g for 15 min at 4°C and pre-cleared with 20 μl pre-washed Streptavidin agarose beads (Invitrogen) at 4°C for at least 1 hour with gentle rotation. Biotinylated RNA (30 μg) was restructured in RNA structure buffer (10 mM Tris pH 7.0, 0.1 M KCl, 10 mM $MgCl_2$) at 90°C for 2 min, immediately transferred on ice and incubated for 2 min, then incubated at room temperature for 20 min to allow proper RNA secondary structure formation. Folded RNA was then mixed with 900 μL of nuclear lysate and tRNA (0.1 mg/ml) at 4°C for 6 hours. Prewashed streptavidin-agarose beads (50 μl) were added into the pre-cleared nuclear lysate, and then incubated for 7 hours at 4°C. At the end of the incubation, the mixture was centrifuged at 1000 g at 4°C for 3 min and the supernatant was discarded. The beads were washed with ice-cold wash buffer (50 mM Tris pH7.4; 150 mM Nacl, 1% NP-40, 0.5% Triton x-100, 1 mM PMSF, 1 mM cocktail, 200 U/ml RNaseOUT) for seven times then boiled with 2× SDS loading buffer. The proteins were detected by Western blot.

RNA-immunoprecipitation (RIP)

For RIP, 1×10^7 A549 or H460 cells were collected and washed for 3 times with DEPC-PBS. Cells were re-suspended in 1 ml ice-cold cytoplasm isolation buffer (10 mM Hepes, 1.5 mM $MgCl_2$, 10 mM KCl, 0.5 mM DTT, 0.05% NP-40) and vortexed at highest speed for 10 sec, followed by incubating on ice for 1 min. Nuclei were harvested at 1000 g for 3 min at 4°C. Pellets were re-suspended in 1 ml freshly prepared RIP buffer (25 mM Tris pH 7.4, 150 mM NaCl, 5 mM EDTA, 0.5 mM DTT, 1% NP40, 0.5% Triton-100, 1 mM PMSF, 1 mM cocktail

100 U/ml RNaseOut), then lysed with sustaining rotation for 30 min at 4°C. The nuclear lysates were harvested by centrifugation at 16,000 g for 15 min at 4°C. Then 2.5 µg of anti-YB-1 antibody or control IgG (Santa Cruz) were added to the supernatant and incubated for 6 hour at 4°C with gentle rotation. 35 µl protein A/G sepharose beads (Santa Cruz) were added to each sample and incubated at 4°C for 6 hours. Beads were harvested at 1000g for 3 min. The beads were washed for seven times. The pellets were then re-suspended in 50 µl lysis buffer supplemented with 5 µl protease K and incubated at 55°C for 30 min, followed by addition of 1 ml TRIzol reagent (Invitrogen). RNA extraction was performed according to manufacturer's instruction, and the abundance of RNAs was detected by real-time RT-PCR.

Protein-RNA complex immunoprecipitation by 2MS2-12MS system

HEK293T cells were transfected with the 2MS2-12MS system plasmids. The cells (1×10^7) were used for each immunoprecipitation assay. 48 hours after transfection, cells were harvested and washed twice with ice-cold DEPC-PBS. Then the cell pellets were re-suspended in 1 mL lysis buffer (20 mM Tris, pH 7.4, 10 mM NaCl, 2 mM EDTA, pH 8.0, 0.5% Triton-100, 1 mM PMSF, 1 mM proteinase inhibitor cocktail, 0.1 mg/ml tRNA, 200 U/ml RNaseOut) and lysed with sustaining rotation for 30 min at 4°C. Cell lysates were obtained by centrifugation at 16,000 g for 15 min at 4°C. 5% of cell lysates were used as input sample. Prewashed Flag-M2 beads (50 µl) were added into the lysates and incubated for 7 hours at 4°C. The beads were collected and washed seven times with wash buffer (50 mM Tris, pH 7.4, 150 mM NaCl, 0.05% Triton-100, 1 mM PMSF, 1 mM proteinase inhibitor cocktail, 0.1 mg/ml tRNA, 200 U/ml RNaseOut). Half of the beads were boiled with 2× SDS loading buffer, and the rest beads were harvested for RNA extraction.

Nuclear and cytoplasmic RNA fractionations

Nuclear and cytoplasmic RNAs were isolated as previous description [2]. Briefly, 1×10^7 cells were harvested by trypsinization and washed twice with DEPC-PBS. Cell pellets were re-suspend in 200 µl lysis buffer (10 mM Tris HCl, pH8.0, 140 mM NaCl, 1.5 mM $MgCl^2$, 0.5% NP40, 2 mM VRC) and incubated on ice for 5 min. Cell lysates were centrifuged at 12000g for 3 min, and the supernatant (cytoplasm fraction) was collected. The remaining pellets (enriched with nuclei) were washed with 160 µl lysis buffer twice and re-suspend in 100 µl lysis buffer. 1 ml TRIzol Reagent was added to the cytoplasm and nuclei fractions, respectively. RNA isolation and RT-PCR were performed as described above.

Colony formation assay

For soft agar assay, the bottom layer was prepared with a 0.6% agarose (Amresco) solution in DMEM with 10% fetal bovine serum in 35 mm plates, and the gel was placed for 30 min at room temperature. 0.5×10^3 A549-luciferace cells with stable expression of control and *CAR10* shRNAs were added into 0.3% top agarose solution diluted with culture medium, then carefully placed on top of the bottom agarose. The plates were incubated at 37°C with 5% CO_2 for 2 weeks, and cell colonies were stained using 0.05% crystal violet (Amresco) and counted. For colony formation on plates, 0.25×10^3 A549-luciferace cells with stable expression of control and *CAR10* shRNAs diluted in culture medium were plated into 6-well plates, and the plates were incubated at 37°C in 5% CO_2 air for 1 week, and culture medium was replaced twice. Finally, cell colonies were stained with 0.05% crystal violet (Amresco) and counted.

Animal studies

The animal studies were approved by the Institutional Review Board of Institute of Zoology, Chinese Academy of Sciences. All animal studies were conducted according to protocols approved by the Animal Ethics Committee of the Institute of Zoology, Chinese Academy of Sciences. Six-week-old female SCID Beige mice were kept in specific pathogen-free (SPF) environment and used for the animal assays. A549-luciferase cells stably expressing control and *CAR10* shRANs were trypsinized and harvested in PBS, then a total volume of 0.1 ml PBS containing 1×10^6 cells were injected into the tail vein of the animals (n=15 for each group). Twenty-five days later, the mice were anesthetized by mixture of oxygen/isoflurane inhalation and positioned with legs fully extended, injected with 150 mg/kg body weight D-luciferin substrate (Gold Biotechnology, St. Louis, MO, USA), and the bioluminescence signals were detected by the IVIS Spectrum Imaging System (Caliper Life Sciences; Hopkinton, MA, USA). Survival of the mice was evaluated from the first day of cell transplantation until death or became moribund, at which time points the mice were sacrificed.

REFERENCES

1. Cozens AL, Yezzi MJ, Kunzelmann K, Ohrui T, Chin L, Eng K, Finkbeiner WE, Widdicombe JH, Gruenert DC. CFTR expression and chloride secretion in polarized immortal human bronchial epithelial cells. Am J Respir Cell Mol Biol. 1994; 10:38-47.
2. Yin QF, Yang L, Zhang Y, Xiang JF, Wu YW, Carmichael GG, Chen LL. Long Noncoding RNAs with snoRNA Ends. Mol Cell 2012; 48:219-230.

Table S1: Differentially expressed lncRNAs in NSCLC tissues. Provided as an Excel file.

See Supplementary File 1

Table S2: Differentially expressed mRNAs in NSCLC tissues. Provided as an Excel file.

See Supplementary File 2

Table S3: Differentially expressed lncRNAs in HPR and CR NSCLCs. Provided as an Excel file.

See Supplementary File 3

Table S4: Differentially expressed mRNAs in HPR and CR NSCLCs. Provided as an Excel file.

See Supplementary File 4

Table S5: Go terms of dysregulated mRNAs. Provided as an Excel file.

See Supplementary File 5

Table S6: Sequences of primers for RT-PCR, real-time PCR, siRNA, and shRNAs

Target	Forward primer (5’→3’)	Reverse primer (5’→3’)
RT-PCR	7	
GAPDH	GAGTCAACGGATTTGGTCGT	GACAAGCTTCCCGTTCTCAG
CAR10	GGGGAAGTTAGCCACAGA	AACCTGGAAGAGCTGAATAG
ACTA1	GGACTTCGAGCAAGAGATGG	AGCACTGTGTTGGCGTACAG
MALAT1	GGTCTTTGGTGGGTTGAACT	TTCCCACCCAGCATTACAGT
12MS	ACGACTCACTATAGGGAGACCCAAG	GATATCAGCTCTAGCATTTAGGTGACACT
Real-time RT-PCR		
GAPDH	GAGTCAACGGATTTGGTCGT	GACAAGCTTCCCGTTCTCAG
CAR10	TCTGCTGGACTTAGGCTGGT	TGCTGCAGTGTGTGGCTATC
YB-1	ACCACAGTATTCCAACCCTCCTG	ATCTTCTTCATTGCCGTCCTCTC
FOXF2	CACTACTGGACCATCGACCCC	CTCACCACGCGGTGGTACAT
EGFR	GACAGCTATGAGATGGAGGAA	GAGTCACCCCTAAATGCCA
siRNA		
CAR10-1	GAGUGACUCAUUCUCCUGUTT	ACAGGAGAAUGAGUCACUCTT
CAR10-2	CAGGUCAAAUCAGGAGGCUTT	AGCCUCCUGAUUUGACCUGTT
YB1-1	GGCGAAGGUUCCCACCUUATT	UAAGGUGGGAACCUUCGCCTT
YB1-2	CGGCAAUGAAGAAGAUAAATT	UUUAUCUUCUUCAUUGCCGTT
FOXF2	GAGCGUCUGUCAGGAUAUUTT	AAUAUCCUGACAGACGCUCTT
NC	UUCUCCGAACGUGUCACGUTT	ACGUGACACGUUCGGAGAATT
shRNAs		
CAR10-1	CCGGCAGAGTGACTCATTCTCCTGTCTCGA<br>GACAGGAGAATGAGTCACTCTGTTTTTG	AATTCAAAAACAGAGTGACTCATTCTCCT<br>GTCTCGAGACAGGAGAATGAGTCACTCTG
CAR10-2	CCGGCAAAGCCAGTGGCTAATACATCTC<br>GAGATGTATTAGCCACTGGCTTTGTTTTTG	AATTCAAAAACAAAGCCAGTGGCTAATAC<br>ATCTCGAGATGTATTAGCCACTGGCTTTG

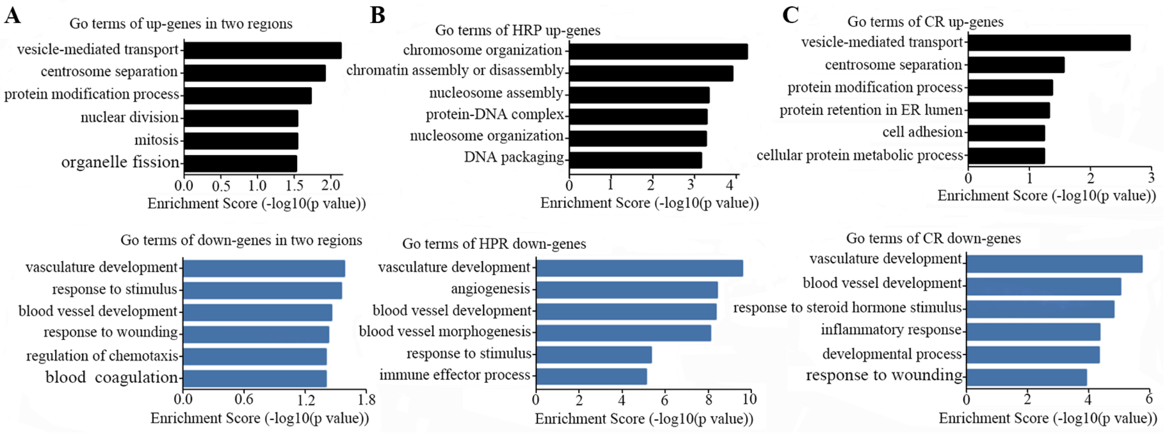

Figure S1: A GO analysis of the altered mRNAs. A. The top 6 GO terms of up-regulated and down-regulated mRNAs in both the HPR and CR NSCLCs, respectively ($p<0.05$). **B.** The top 6 GO terms of up-regulated and down-regulated mRNAs in HPR patients, respectively ($p<0.05$). **C.** The top 6 GO terms of up-regulated and down-regulated mRNAs in CR cases, respectively ($p<0.05$).

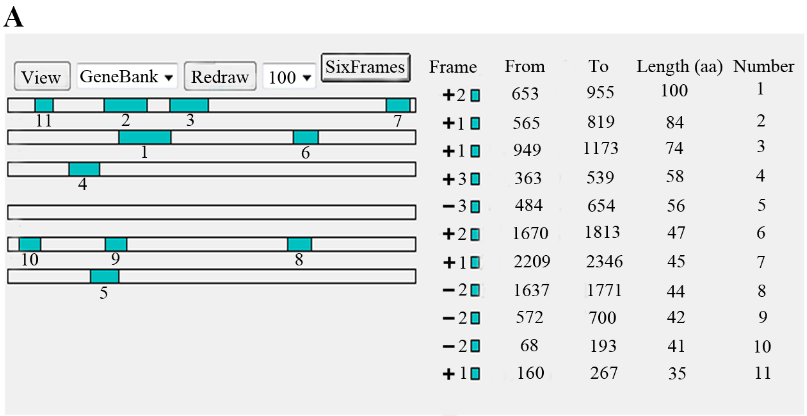

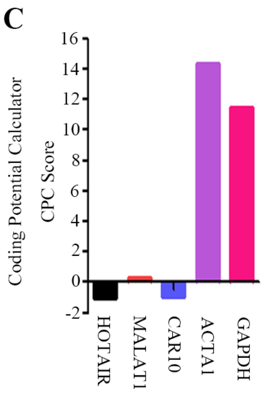

B

No.of ATG from 5'end	Reliability (%)	Frame	Identity to Kozak rule A/GXXATGG	Start (bp)	Finish (bp)	ORF Length (aa)	Stop codon found?
15	23	2	cXXATGc	653	952	100	YES
20	18	1	tXXATGc	949	1170	74	YES
13	12	1	cXXATGt	565	816	84	YES
32	10	2	AXXATGa	1670	1810	47	YES
14	8	1	cXXATGa	589	816	76	YES

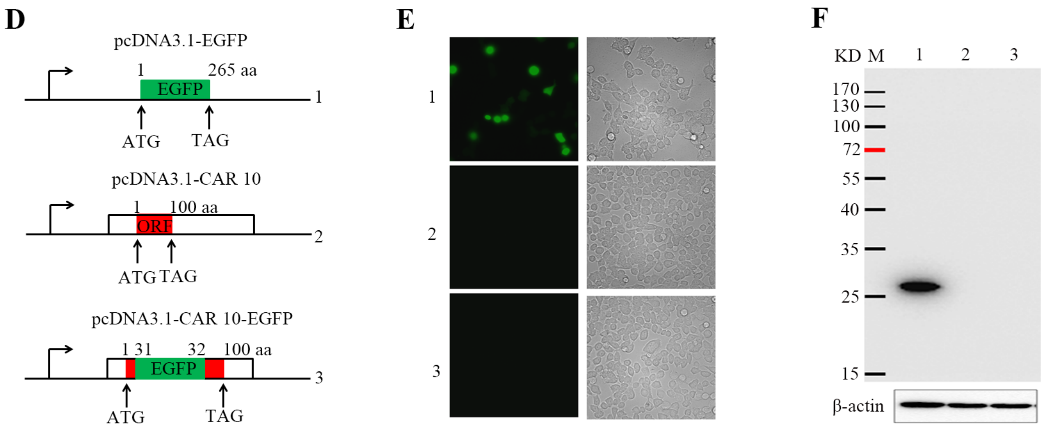

Figure S2: *CAR10* has no protein coding potential. A. ORF analysis of *CAR10*. **B.** Analysis of Kozak's motifs of *CAR10*. **C.** The coding potential calculator (CPC) score of the indicated lncRNAs and protein coding genes. **D.** Schematic representation of constructions for pcDNA3.1-EGFP, pcDNA3.1-*CAR10* and pcDNA3.1-*CAR10*-EGFP. **E.** The plasmids were transfected into HEK293T cells, and the green fluorescence was detected by fluorescence microscope 48 hours after transfection. **F.** HEK293T cell transfected with plasmids mentioned above were lysed 48 hours after transfection, and the lysates were subjected to Western blotting.
